# Visual Quality in Acute Retinal Pigment Epitheliitis: A Case Report

**DOI:** 10.3390/reports9020149

**Published:** 2026-05-12

**Authors:** Francisco de Asís Bartol-Puyal, Carlos Santana Plata, Carmen Bilbao Porta, Claudia Sanz Pozo, Silvia Méndez-Martínez, Luis Pablo

**Affiliations:** 1 Ophthalomology Department, Miguel Servet University Hospital, 50009 Zaragoza, Spain; 2 Miguel Serveet Ophthalmology Research Group (GIMSO), Aragón Institute for Health Research (IIS Aragón), 50009 Zaragoza, Spain; 3 Department of Surgery, Universidad de Zaragoza, 50009 Zaragoza, Spain; 4 Biotech Vision SLP, Spin-Off Company, Universidad de Zaragoza, 50011 Zaragoza, Spain

**Keywords:** acute retinal pigment epitheliitis, contrast sensitivity, color perception, visual quality

## Abstract

**Background and Clinical Significance:** Acute retinal pigment epitheliitis is a retinal disorder considered as part of a larger group named idiopathic choroidopathies. Little gray round macular lesions at the retinal pigment epithelium can be found, which are self-limited, resolving within 6–12 weeks. It can decrease best corrected visual acuity (BCVA), but visual quality has not been studied yet. **Case Presentation:** A 17-year-old Caucasian boy who came to our ophthalmology department and presented with acute retinal pigment epitheliitis in his right eye (OD). BCVA under mesopic lighting was 0.18 logMAR in his OD and −0.18 in his OS. With a neutral density filter, it was 0.52 and 0.04, respectively. Contrast sensitivity was assessed with the CSV-1000E test, but OD outcomes were worse only in the case of mesopic lighting. Chromatic discrimination was assessed with the Farnsworth–Munsell 100 test and revealed marked impairment of both red-green and yellow-blue axes. No central scotoma was detected on a 10.2 visual field, nor was any halo perception detected with the Halometer test. **Conclusions:** BCVA under low illumination and color perception in the yellow-blue axis may be affected in patients with acute retinal pigment epitheliitis to a greater extent than previously described. Contrast sensitivity may also be altered, but to a lesser extent.

## 1. Introduction and Clinical Significance

Acute retinal pigment epitheliitis (ARPE) is a retinal disorder considered as part of a larger group referred to as idiopathic choroidopathies. It was first described in 1972 by Krill and Deutman [1], and it usually affects young patients between 20 and 40 years of age. Both males and females are equally affected. It usually affects one eye, but some bilateral cases have been reported as well [2]. Its pathophysiology is unknown, but an immune response against retinal pigment epithelium (RPE) [2] or against neurosensory retina could be involved [3,4], and it has been associated with viral infections [5,6]. It is a rare condition, and no multicenter studies have been performed so far. This leads to a lack of consensus on the definition of ARPE [7].

Symptoms at the beginning may vary from asymptomatic to blurred vision and central scotoma [2]. The only lesions that can be detected on fundus examination are macular pigment stippling surrounded by hypopigmented halos [2]. Some hyperreflective lesions can be observed on optical coherence tomography (OCT) slabs, in the outer nuclear layer (ONL) and photoreceptor layer (PRL), with disruption in the external limiting membrane (ELM) and the ellipsoid zone (EZ) [2,8]. RPE undulation or irregularity is another possible finding in OCT [9]. The restoration of lesions on OCT follows this sequence: a decrease in the height of the hyper-reflective lesion with ELM returning to its normal position with irregularity, then complete disappearance of the hyper-reflective lesion, afterwards restoration of the ELM, then restoration of EZ, and finally restoration of the interdigitation zone [4].

Fluorescein angiography reveals transmission hyperfluorescence, and indocyanine green angiography shows the pathognomonic late halo-like or focal hyperfluorescence [8].

ARPE lesions are self-limited, with spontaneous recovery in 6–12 weeks [2]. However, subsequent recurrences have been described in the literature [10,11]. It is not necessary to treat, but corticosteroids have been used in cases of severe visual impairment. However, the role of corticosteroids as a treatment is yet to be defined [4].

Due to overlapping features, differential diagnosis should include multiple evanescent white dot syndromes and acute posterior multifocal placoid pigment epitheliopathy [3].

Despite multiple case reports in the scientific literature, visual quality has not been evaluated during the acute phase and afterwards, as far as we know. Since central RPE and PRL are disrupted in OCT, an alteration should be expected in visual field (VF), best corrected visual acuity (BCVA) under low luminance, color perception, contrast sensitivity (CS), or halo vision, among others.

Herein, we present visual-quality outcomes in a case of a young patient with unilateral recurrent ARPE.

## 2. Case Presentation

A 17-year-old Caucasian boy presented with sudden blurred vision in his right eye (OD) in 2024. He had no relevant medical history and was not taking any medication. He did not refer to other symptoms, neither systemic nor ophthalmological. Three years before, he had already come to the department because of similar symptoms in the same eye, but no diagnosis had been made, which resolved spontaneously in about a month.

**Figure 1:**
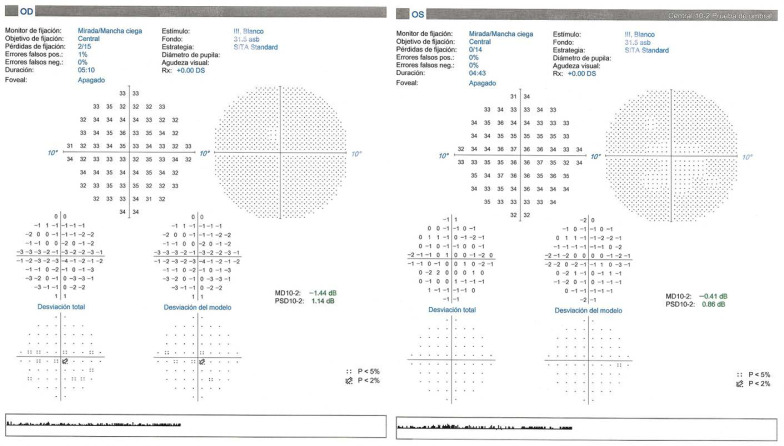
Visual field during active phase. OD: Right Eye; OS: Left Eye; Central 10-2 Prueba de umbral: Central 10-2 Threshold Test; Monitor de fijación: Fixation Monitor; Mirada/Mancha ciega: Gaze/Blind spot; Objetivo de fijación: Fixation Target; Central: Central; Pérdidas de fijación: Fixation Losses; Errores falsos pos.: False Positive Errors; Errores falsos neg.: False Negative Errors; Duración: Duration; Foveal: Foveal; Apagado: Off; Estímulo: Stimulus; III, Blanco: III, White; Fondo: Background; Estrategia: Strategy; SITA Standard: SITA Standard; Diámetro de pupila: Pupil Diameter; Agudeza visual: Visual Acuity; Desviación total: Total Deviation; Desviación del modelo: Pattern Deviation.

The refraction in his OD was sphere (S) 0 D, cylinder (C) −0.75 at 174°. In his left eye (OS), it was S 0, C 0. Axial length (AL) was measured with IOLmaster 500 (Carl Zeiss Meditec, Jena, Germany), and it was 23.07 mm in his OD and 23.09 mm in his OS. BCVA was measured with ETDRS charts at a 4 m distance under mesopic lighting conditions (3 cd/m^2^) in a controlled lighting cabinet after 5 min of light adaptation. It was 0.18 in logMAR units (20/30 in Snellen) in his OD, and −0.18 (20/13 in Snellen) in his OS. When adding a neutral density filter, it was 0.52 in logMAR units (20/66 in Snellen) in his OD, and 0.04 (20/22 in Snellen) in his OS. The filter used was the Kodak Wratten 2.0 Neutral Density Filter (Eastman Kodak Company, Rochester, NY, USA). It reduces light intensity and transmits 1% of the incoming light. A 10-2 VF was performed using Humphrey Zeiss HFA 3 (Carl Zeiss Meditec, Jena, Germany), and it revealed no central scotoma. VF is shown in Figure 1.

CS was measured with the CSV-1000E test (VectorVision, Greenville, OH, USA) at a 2.5 m distance with distance optical correction. It measures CS in the following spatial frequencies: 3, 6, 12 and 18 cycles/degree. This test was first performed under mesopic lighting conditions, then adding a neutral density filter, and finally with no filter but with glare (40 cd/m^2^). Reference values were taken from previously published studies with the same examination conditions [12]. Outcomes in OD were slightly lower than OS, but no clear differences were found in comparison with reference values. These outcomes are displayed in Table 1.

Chromatic discrimination was assessed with the Farnsworth–Munsell 100 test monocularly at a 40 cm distance and under mesopic lighting. Outcomes in his OD were classified as abnormal, but they did not fit into protanomaly, deutanomaly or tritanomaly spectra. As expected, the outcomes in his OS were classified as within normal range. The outcomes are displayed in Table 2. Reference values were taken from the scientific literature [13].

Halo perception was evaluated with Halometer (Universidad de Granada, Granada, Spain) under scotopia (0 cd/m^2^) at a 40 cm distance monocularly. Five minutes were allowed for light adaptation to scotopia. The test was performed with the following settings: 20 px of main stimulus radius, 2 px of peripheral stimulus radius, 30 px of maximum radius, 3 stimuli per semiaxis, 12 semiaxis, 10 s of darkness, 5 s of maximum stimulus, 1 s of exposure, and refresh from 2 to 3 s. Outcomes for both eyes (OU) were the same and considered as normal. Linear discrimination indexes were 1, quadratic discrimination indexes were 1, linear alteration indexes were 0, and quadratic alteration indexes were 0 in OU.

**Figure 2:**
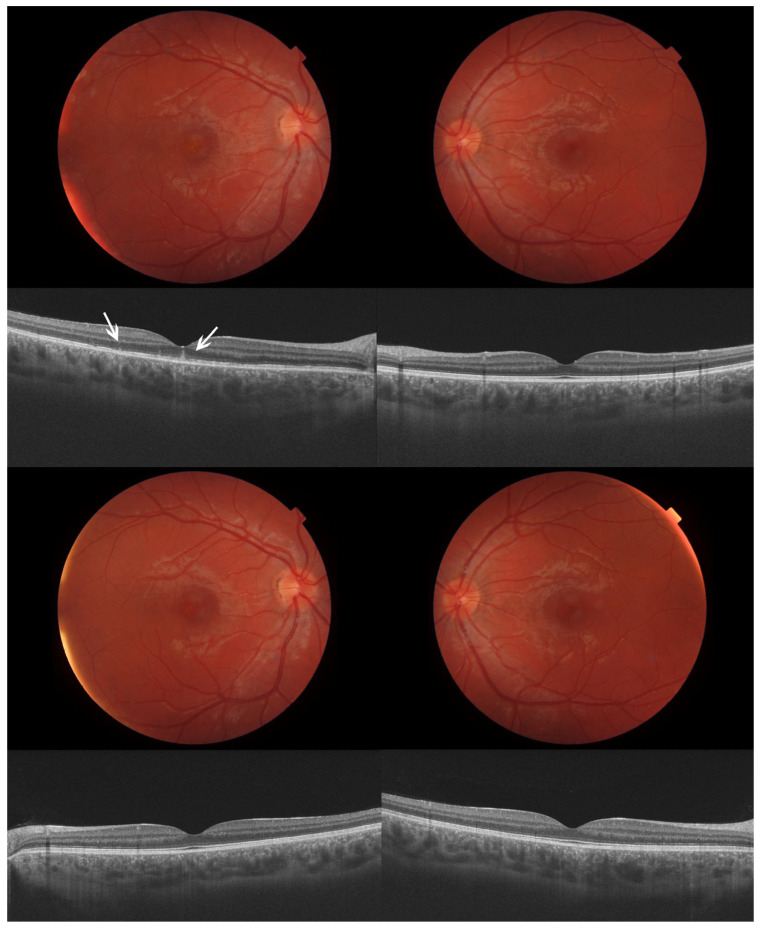
Composition of multimodal imaging in both eyes. OD on the left, OS on the right side. (**First row**), fundus pictures during the active phase. (**Second row**), SS-OCT slabs during the active phase. (**Third row**), fundus pictures after recovery. (**Fourth row**), SS-OCT slabs in the chronic phase.

Slit-lamp examination revealed no alterations in the anterior segment. Intraocular pressure (IOP) measured with Goldmann tonometry was 14 mmHg in OU. Afterwards, his pupils were dilated with topical tropicamide. No abnormal findings were detected in his OS, but abnormal findings were observed in his OD. Small grayish spots were found in the fovea. Figure 2 displays fundus pictures and OCT slabs.

**Figure 3:**
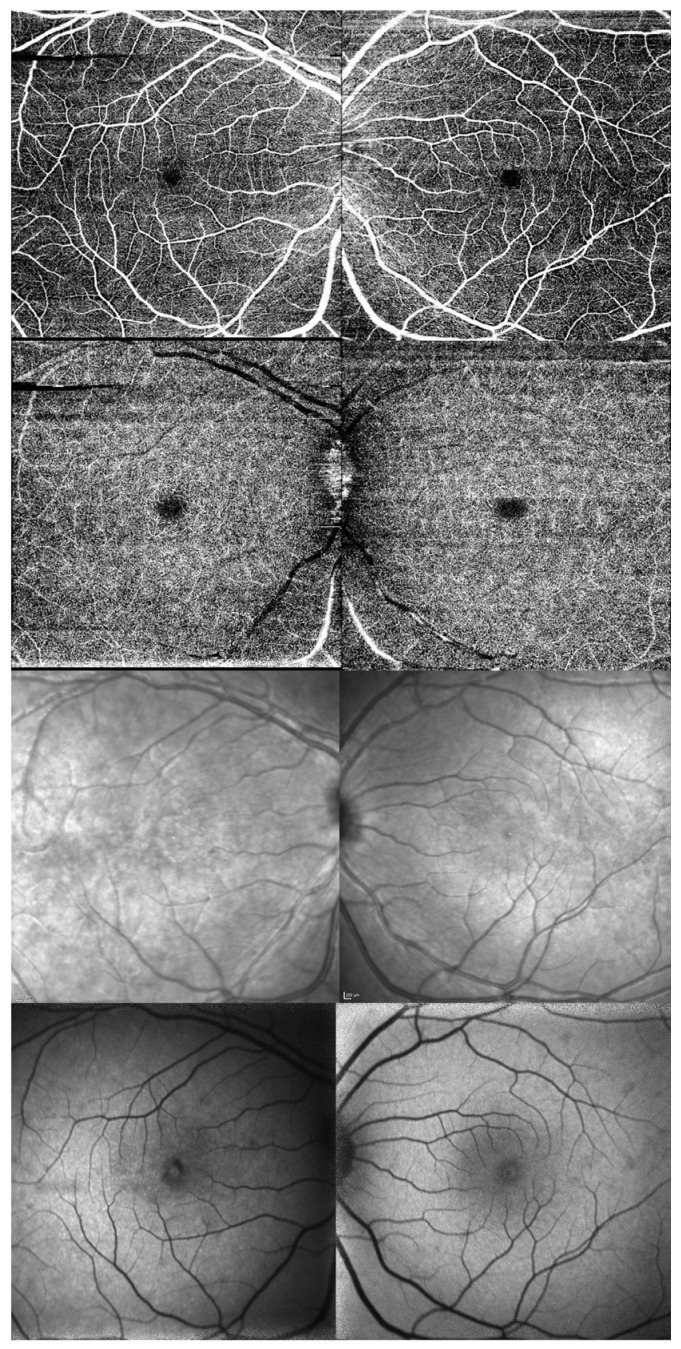
Composition of multimodal imaging in both eyes during the active phase. OD on the left, OS on the right. (**First row**), superficial capillary plexus in OCTA. (**Second row**), deep capillary plexus in OCTA. (**Third row**), infrared pictures. Fourth row, autofluorescence pictures.

These foveal lesions were better assessed with spectral-domain (SD) OCT Spectralis (Heidelberg Engineering, Heidelberg, Germany) and with swept-source (SS) OCT DRI Triton (Topcon Corporation, Tokyo, Japan). A hyper-reflective lesion emerging from the RPE, disrupting the EZ and the ELM, and reaching the ONL was observed in the fovea. A certain level of PRL atrophy was present, with hypertransmission to the choroid, but some mild degree was already present since the previous episode three years before. Choroidal thickness (CT) was around 500 μm in the fovea in OU, but without pachyvessels. Figure 3 shows multimodal imaging.

No alterations were detected on OCT-angiography (OCTA) Triton. Retinal vasculature was normal in OU. Neither were any disturbances detected using infrared reflectance (815 nm), nor in autofluorescence (486 nm) (OCT Spectralis). Figure 3 shows multimodal imaging.

Upon reviewing previous examinations three years before, the same retinal spots had appeared on OCT in his OD when the patient came with decreased BCVA. In the meantime, these spontaneously disappeared, while his BCVA recovered with no treatment. A certain level of PRL atrophy remained since then.

Differential diagnosis included idiopathic choroidopathy, posterior uveitis, retinal dystrophy, and retinal toxicity secondary to drugs or medications. Final diagnosis was ARPE because the signs were not typical for uveitis, complete resolution from the first episode ruled out the possibility of a retinal dystrophy, and he was not under treatment with any medication.

Four months later, the patient reported an improvement in his vision, and he was reevaluated with the same tests and by the same examiner. No treatment was administered during this time period. There were no differences in BCVA or the other visual-quality tests between OU. These outcomes are presented in their respective tables, under the name of ‘chronic phase’. Foveal spots disappeared, as well as the disruption in ELM-PRL detected in OCT. CT remained the same in OU. These pictures are presented together with those of the acute phase. Fundus pictures and OCT slabs are displayed in Figure 2.

## 3. Discussion

ARPE is an idiopathic condition that mainly affects young patients, causing decreased BCVA. It resolves spontaneously in 6–12 weeks, although minor alterations may persist. As a consequence of retinal lesions, visual quality should be expected to become altered, too. Nevertheless, this has not been studied yet, as far as we know. Herein, we present a case of a patient with ARPE in whom we performed different tests to assess visual quality. Furthermore, this is the third reported recurrent case to our knowledge [10,11].

According to other authors, this clinical entity is variable in the extent of the neurosensory retina involvement [14]. In this case, retinal lesions are rather small in comparison with other cases. This should be taken into account when evaluating visual quality tests in this case.

OCT revealed foveal affection with a hyper-reflective lesion from RPE to ONL, which could rather be classified as ARPE type 2 according to the classification proposed by Ahmed-Balestra et al. [8], but the lesion was unilateral, and the patient experienced complete restoration of BCVA. The spontaneous restoration of this lesion in OCT was complete four months after the acute phase, similar to previously described cases of ARPE. The patient did not receive any kind of treatment because of the lack of evidence and consensus in the scientific literature.

Some previous reported cases showed disturbances in choriocapillaris, together with RPE and neurosensory retinal disturbances [10]. On the contrary, we could not detect any vascular disorder in the retina or choriocapillaris using OCTA.

ARPE lesions cannot be visualized using blue or green autofluorescence, but they can in infrared pictures [3]. However, we could not detect any disturbances using infrared wavelength.

BCVA decrease was greater in low mesopic conditions. This implies that the patient experiences lower vision in poorly illuminated places. This highlights the importance of measuring BCVA under low luminance in order to assess visual function in a patient’s everyday life. It is also an earlier clinical marker of changes in the central retina [15]. In contrast to previous research [2,9], we detected no central scotoma in VF analysis. This is likely due to the small size of the lesions, and possibly a microperimetry might reveal some tiny scotomas.

CS showed a certain lowering under mesopic lighting, but it was not present in all spatial frequencies and examination conditions. Additionally, all values could be considered within normal ranges. Relatively decreased CS outcomes recovered four months after the acute phase.

Color perception was deeply altered in OD. Both red-green and blue-yellow axes were affected, but it was the latter in which the alteration was even greater. Nonetheless, this alteration could not be classified as protanomaly, deutanomaly or tritanomaly. Some time after the acute phase, this alteration recovered completely. Dyschromatopsia may appear early in pathologies such as glaucoma [16] or retinal dystrophies [17].

The patient did not suffer from halo perception. This is more typical for patients implanted with multifocal intraocular lenses [18,19], but it can appear in other retinal disorders as well [20].

This disease is uncommon, and this is the third reported case of recurrence. In addition, this is the first time that a deep assessment of visual quality has been performed in a patient with this clinical entity. No clear explanation has yet been provided for its recurrence. The immune system and viral antigens might be the underlying cause, but this is only a theory [10]. This patient had no flu-like symptoms at all in both the first and second active phases.

Future research should be performed in these patients to evaluate visual quality during the acute phase. Although this is the first time that such a deep visual quality assessment has been reported, larger studies should be performed to confirm our findings.

## 4. Conclusions

In conclusion, visual function may be impaired in patients with ARPE to a greater extent than previously described, especially BCVA under low illumination and color perception on the yellow-blue axis. These abnormal findings may recover completely after the acute phase and without treatment.

## Figures and Tables

**Table 1:** CSV-1000E test outcomes, in decimal scale.

Spatial Frequency	Phase	Mesopic			Mesopic + Neutral Filter			Mesopic + Glare		
		Ref	OD	OS	Ref	OD	OS	Ref	OD	OS
3 cycles/degree	Acute	1.59	1.63	1.93	1.56	1.63	1.63	1.44	1.63	1.78
	Chronic		1.78	1.78		1.63	1.78		1.63	1.34
6 cycles/degree	Acute	1.83	1.84	2.14	1.44	1.55	1.38	1.40	1.84	1.70
	Chronic		1.99	1.70		1.70	1.38		1.70	1.55
12 cycles/degree	Acute	1.41	1.99	1.99	0.95	1.08	1.08	0.79	0.91	1.54
	Chronic		1.54	1.99		1.08	1.08		1.08	0.91
18 cycles/degree	Acute	0.99	0.81	1.55	0.50	0.81	0.64	0.42	1.10	1.25
	Chronic		1.10	1.40		0.64	0.64		0.81	0.64

Ref: reference.

**Table 2:** Farnsworth–Munsell 100 outcomes.

	Phase	Ref	OD	OS
Total error score	Acute	120	216	40
	Chronic		120	40
Partial error score red-green	Acute	70	82	28
	Chronic		40	20
Partial error score blue-yellow	Acute	50	134	12
	Chronic		80	14
Confusion index	Acute	1.00	2.61	1.26
	Chronic		1.02	1.03
Scatter index	Acute	1.20	1.66	1.29
	Chronic		1.23	1.29
Confusion angle, ^o^	Acute	-	60.6	41.9
	Chronic		59.3	60.0

Ref: reference.

## Data Availability

The original contributions presented in this study are included in the article. Further inquiries can be directed to the corresponding author.

## Abbreviations

The following abbreviations are used in this manuscript:

ARPE	Acute Retinal Pigment Epitheliitis
AL	Axial Length
BCVA	Best Corrected Visual Acuity
C	Cylinder
CS	Contrast Sensitivity
ELM	External limiting membrane
EZ	Ellipsoid Zone
OCT	Optical Coherence Tomography
OCTA	Optical Coherence Tomography-Angiography
OD	Right eye (Oculus Dexter)
ONL	Outer Nuclear Layer
OS	Left eye (Oculus Sinister)
OU	Both eyes (Oculus Uterque)
PRL	Photoreceptor Layer
RPE	Retinal pigment epithelium
S	Sphere
SS	Swept-Source
VF	Visual Field
